# Decrease in TSH Receptor Autoantibodies during Antithyroid Treatment: Relationship with a Long Noncoding Heg RNA and Cdk1 mRNA in Mononuclear Cells

**DOI:** 10.5402/2011/287052

**Published:** 2011-07-06

**Authors:** Niels Juel Christensen, Gurli Habekost, Palle Bratholm

**Affiliations:** Endocrine Research Laboratory, Medical Department O, Herlev Hospital, University of Copenhagen, 2730 Herlev, Denmark

## Abstract

We have previously shown that a long noncoding RNA transcript Heg is negatively correlated with TSH receptor autoantibodies (TRAb) in patients with untreated Graves' disease and with CD14 mRNA in treated patients and controls. Thus patients with high concentrations of Heg RNA have low levels of TRAb or CD14 mRNA, respectively. Here we show that an additional factor, gene expression of Cdk1 in mononuclear cells, is positively related to concentrations of TRAb in patients with untreated Graves' disease. Cdk1 mRNA is very important for regulation of cell cycle activity. It is well known that TRAb decrease significantly during treatment with antithyroid drugs. This decrease during treatment cannot be explained by Heg RNA, which remains unchanged. Cdk1 mRNA decreased significantly during treatment to values below values obtained in normal subjects. Thus both Heg RNA and Cdk1 mRNA may influence the level of TSH receptor autoantibodies but by different mechanisms.

## 1. Introduction

We have previously shown that a long non-coding RNA transcript Heg in MNC (GenBank EU137727) is negatively correlated with TRAb in patients with early and untreated Graves' disease and with CD14 mRNA in treated patients and in normal subjects. Furthermore, transfection studies with fragments of Heg (exogenous Heg) decreased CD14 mRNA [1] and increased TLR7 and INF-*γ* mRNA in MNC (unpublished results). Low CD14 values may reduce IL-12 and activation of monocyte-dendritic cell signaling and autoantibody production [2]. It cannot be excluded that Heg may be protein-coding and the existence of open reading frames within the Heg sequence warrants further study [3]. TRAb decrease during treatment with antithyroid drugs. The mechanism has not been clarified [4–8]. The primary aim of the present study was therefore to examine if changes in TRAb during antithyroid treatment were related to changes in Heg RNA and CDl4 mRNA. Secondly, we wanted to examine if TRAb was related to Cdk1 mRNA, which is an important factor for regulation of cell cycle activity [9].

## 2. Subjects

All subjects gave informed consent. The study protocol was approved by the Ethics Committee of Copenhagen County and was in compliance with the declaration of Helsinki II. Samples were obtained from three main study groups.

1. Seventeen patients with Graves' disease were studied at the time of diagnosis before treatment with antithyroid drugs had begun. The mean age was 48 years (range from 35 to 67 years). Sixteen were females and one a male. The median level of TRAb was 11.4 IU/l (range of 5.8 (25%)–16.1 (75%)).
2. Twenty patients with Graves' disease were studied after treatment had been initiated. The mean age was 41 years. Fifteen were females and 5 were males. mRNA levels were not measured in these patients before treatment was started but at a followup after median 11 months of treatment (range of 4 (25%)–17.5 (75%)). At the time of examination thyroid hormones were normalized in approximately half of the subjects and 11 subjects had minor elevations in T3 values or suppressed TSH levels. In the majority of patients, the TRAb level decreased from the time of diagnosis to the time of the study. Six of the patients were treated with PTU and 14 patients were treated with Thiamazol. TRAb were available both before and after treatment had been initiated, but no gene expression levels were available before treatment had started. The median TRAb level before treatment was started was 13.5 IU/l (range 9–21.5) and 6.5 (range 2.5–20) during treatment at the time of the present investigation (*P* < 0.004).
3. Eighteen normal subjects were all healthy with a mean age of 45 years. Ten were females and eight males.

## 3. Methods

Methods applied have been described previously [1, 10] and will only briefly be mentioned here.

### 3.1. Isolation of RNA

RNA was isolated from 5 × 10^6^ MNC. For isolation of RNA, we applied the Qiaamp Blood Mini Kit (Qiagen Gmbh, Hilden, Germany). Both total RNA and DNA concentrations were determined.

### 3.2. Quantification of Heg RNA, CD14 mRNA, and Cdk1 mRNA in MNC

mRNA was quantified by RT-PCR-HPLC [1, 10]. HPLC was applied to separate the peak value of the specific standard and the mRNA to be measured.

### 3.3. Primers and Construction of Internal Standards

The oligonucleotide primers were synthesized at DNA Technology (Aarhus, Denmark) or by MWG (Germany).

For quantification of the *Heg RNA,* we used the following set of primers:

Upper primer 5′-GCG CCT GGT ATT AGA T-3′
Lower Primer 5′-CTT TTT CAT ATC CCG ATC TT-3′

*CD14 mRNA*

Upper primer 5′-TTC TAA AGC GCG TCG ATG C-3′
Lower primer 5′-ATT CTG GAT GGC CGG GAA CTT-3′

*Cdk1 mRNA*

Upper primer 5′-CTT GGA AAT TGA GCG GAG AG-3′
Lower primer 5'-TAC TGA CCA GGA GGG ATA GA-3′

An internal standard RNA for Heg and other mRNAs were constructed using the above-mentioned sets of primers and the PCR-MIMIC construction kit from Clontech (BD Biosciences Clontech, Palo Alto, CA). The size of the internal standard (number of bases) was designed to be 239 for Heg, 240 for CD14, and 240 for Cdk1. Amounts of internal RNA standard added to RT-PCR were for Heg 0.12 amol, for CD14 4.36 amol, and for Cdk1 19.5 zmol.

### 3.4. Quantification of PCR Products by HPLC

The HPLC system consisted of a TSK DEAE-NPR column (4.6 mm I.D. × 35 mm) thermostated at 30°C. The pump was a WATERS Model 616 gradient pump controlled by Empower software, which was also applied for data acquisition and processing. Detection was by an Applied Biosystems Model 759A UV-detector at 254 nm. The PCR product was quantified relative to the internal standard using areas and corrected for different sizes of the two products. mRNA concentrations were expressed as amol mRNA/*μ*g DNA or as zmol mRNA/*μ*g DNA.

### 3.5. Validation of the Technique and Reproducibility

These results have been described previously [1].

### 3.6. Hormones

Thyroid hormones and TRAb were measured as described earlier [1].

### 3.7. Statistics

The statistical analysis was performed by the SigmaStat 3.1.1 (SPSS Inc., Chicago, IL). The following statistical tests were applied: Student's *t*-test, one-way ANOVA, linear regression analysis, and multiple regression analysis. A *P* value <0.05 was considered significant.

## 4. Results

Multiple regression analysis was performed in untreated patients with Graves' disease with log TRAb as the dependent variable and Heg RNA amol/*μ*g DNA and log Cdk1 mRNA zmol/*μ*g DNA as independent variables. Heg RNA was as reported previously negatively correlated with log TRAb (*P* < 0.001). Cdk1 mRNA was positively related to TRAb (*P* < 0.002), and including Cdk1 RNA in the regression analysis increased the *r* value (numerically) from −0.61 to −0.83 (*r* = − 0.83; *P* < 0.001):

(1) $$\begin{array}{cc} \log_{10}\text{TRAb}\;\text{IU}/\text{l} & =1.209-\left(12.652\times \text{Heg}\;\text{RNA}\;\text{amol}\right)\\ & \;+\left(0.986\times \log_{10}\text{Cdk}1\;\text{mRNA}\;\text{zmol}\right). \end{array}$$

**Figure 1:**
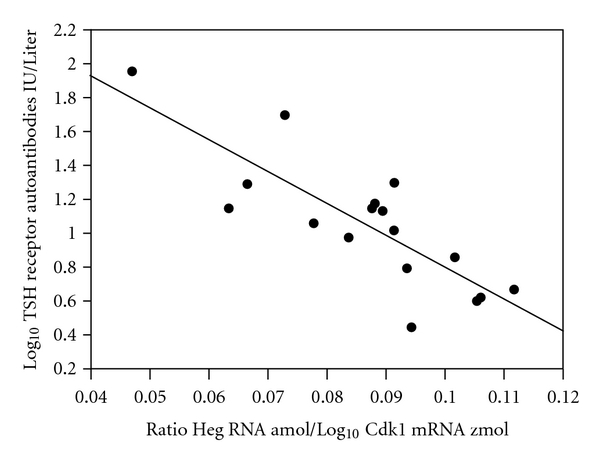
The relationship between log_10_ TSH receptor autoantibodies (TRAb) IU/Liter and the ratio Heg RNA amol per *μ*g DNA/Log_10_ Cdk1 mRNA zmol per *μ*g DNA. *r* = −0.82; *P* < 0.001.

Figure 1 shows the negative relationship observed between log TRAb and the ratio Heg RNA/Log Cdk1 mRNA (*r* = − 0.82; *P* < 0.001). Note that Cdk1 RNA was only related to TRAb provided Heg RNA was included in the statistical analysis. No relationship was established between Cd14 mRNA and Cdk1 mRNA in treated patients with Graves' disease or in normal controls. 

TRAb concentrations but not Heg RNA decreased approximately 50% (from a median level of 13.5 to 6.5 IU/l; *P* < 0.004) during treatment in the 20 patients with Graves' disease. Concentrations of Cdk1 mRNA were significantly reduced in this group of patients as compared with untreated patients and normal subjects. (Table 1; ANOVA *P* < 0.001). The calculated TRAb values obtained from the regression line above after an assumed reduction in Cdk1 mRNA values of approximately 50% also resulted in a decrease in TRAb of 50%. Note that the Cdk1 mRNA decreased below the level observed in normal subjects, and levels were not different in untreated patients and controls. 

## 5. Discussion

Patients with high Heg RNA concentrations had low TRAb values or CD14 mRNA values in untreated patients with Graves' disease and in treated patients and controls, respectively [1]. The present study showed, that decrements in TRAb in treated patients with Graves' disease could not be related to changes in Heg RNA. In patients with untreated Graves' disease, multiple regression analysis with Heg RNA and Cdk1 mRNA as independent variables showed that Cdk1 mRNA values were positively related to TRAB. The *r* value increased from −0.61 to −0.83 as compared to simple regression analysis with Heg RNA as the variable. The main finding in the present study is, however, the significant decrease in Cdk1 mRNA in treated patients with Graves' disease as compared with the other two groups. This may suggest that the decrease in TRAb during antithyroid treatment was related to a decrease in cell cycle activity. Note, however, that the concentration of Cdk1 mRNA was not different in untreated patients and controls, suggesting that the decrease in Cdk1 values during treatment was a specific effect of antithyroid drugs and not due to decrements in thyroid hormone concentrations. Further studies are required to establish the mechanism by which changes in cell cycle activity and antithyroid drugs may decrease TRAb. Our results suggest that there are two different factors, which may influence the level of TRAb. The mechanism is likely to be different. Transfection studies have shown that Heg RNA may reduce gene expression of CD14 mRNA probably by activating TLR7 and INF-*γ*. This may reduce IL-12 and decrease activation of monocyte-dendritic cell signaling. There is no reason to assume, that this effect is specific for patients with Graves' disease. The decrease in CDk1 mRNA and in TRAb is likely to be a specific effect of antithyroid drug treatments, and at present it is unclear if this effect is seen only in patients with Graves' disease. Clearly further studies are required to elucidate the mechanism by which Cdk1 and Heg may regulate TRAb concentrations.

## 6. Conclusion

The present study suggests that two different factors may regulate concentrations of TRAb. A noncoding RNA transcript Heg is negatively related to TRAb in untreated patients with Graves' disease and to CD14 mRNA in treated patients and controls. Transfection experiments have also shown that Heg RNA decreased Cd14 mRNA. The decrease in TRAb observed during antithyroid treatment cannot be explained by Heg RNA, but may be due to a decrease in Cdk1 mRNA, which is important for regulation of cell cycle activity. Gene expression of Cdk1 decreases during treatment to levels below values observed in normal subjects, suggesting that this is a pharmacological effect of antithyroid drugs.

## Figures and Tables

**Table 1:** Cdk1 mRNA concentrations expressed in zmol/*μ*g DNA (median and 25% and 75% ranges) in untreated and treated patients with Graves' disease and in controls.

Untreated patients	Treated patients	Normal subjects
33 (22 to 39)	13 (10–17)*	27 (18–34)

*Significantly different from the two other groups (*P* < 0.001).

## Abbreviations

MNC: Mononuclear cells
TRAb: TSH receptor autoantibodies.
